# Supplementary material for: DNAJB6 mutants display toxic gain of function through unregulated interaction with Hsp70 chaperones
Source: Nat Commun. 2023 Nov 3;14:7066. doi: 10.1038/s41467-023-42735-z (PMC10624832; doi:10.1038/s41467-023-42735-z)
Supplement: Supplementary file 1 — Supplementary Information [file 41467_2023_42735_MOESM1_ESM.pdf]

Supplementary Material

for

DNAJB6 mutants display toxic gain of function through unregulated interaction with Hsp70 chaperones

Meital Abayev-Avraham[1], Yehuda Salzberg[2], Dar Gliksberg[1], Meital Oren-Suissa[2], and Rina Rosenzweig[1,*]

[1]Department of Chemical and Structural Biology, Weizmann Institute of Science, Rehovot, 761000, Israel.
[2]Department of Brain Sciences, Weizmann Institute of Science, Rehovot, 761000, Israel.

[*]rina.rosenzweig@weizmann.ac.il

This file includes:

Supplementary Figs. 1-10

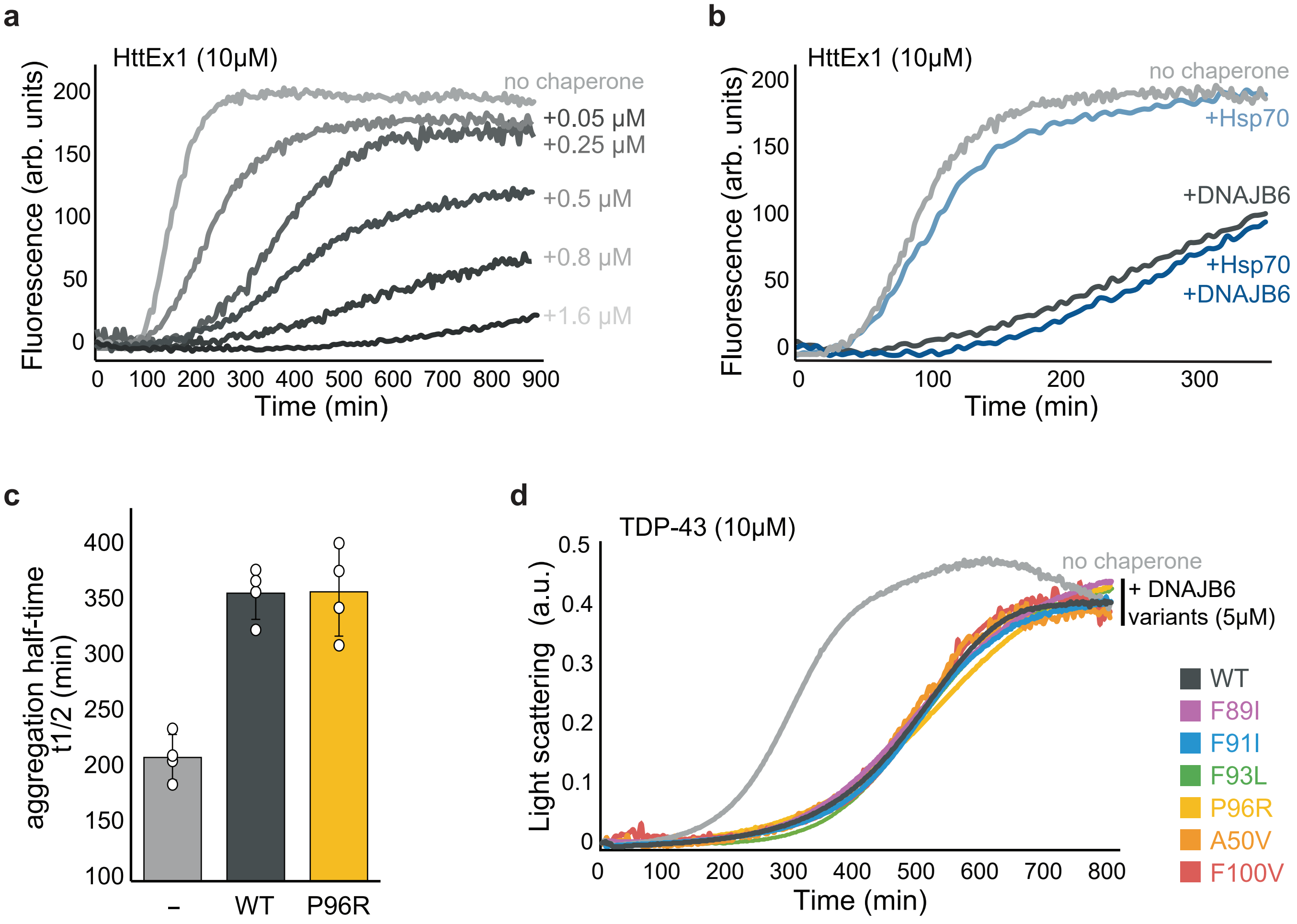

Supplementary Figure 1. LGMDD1 DNAJB6 mutants efficiently suppress protein aggregation
(a) Aggregation of 10 μM HTT_{Ex1}-Q48 alone (light gray), and in the presence of increasing concentrations of WT DNAJB6 (0.05-1.6 μM; light to dark gray). **(b)** The effect of Hsp70 chaperone on HTT_{Ex1}-Q48 aggregation. Addition of Hsp70 did not significantly affect the aggregation prevention activity of DNAJB6 (dark blue). Hsp70 alone showed only a modest aggregation prevention activity (light blue). **(c)** The effect of DNAJB6 WT and P96R disease mutant on HTT^{Ex1}-Q48 aggregation half times. Data represents mean values ± s.d (n=4 independent experiments). **(d)** The liquid to solid transition of 10 μM TDP-43 alone (light gray), and in the presence of 5 μM of WT DNAJB6 (dark gray) or LGMDD1 disease mutants (colored). The mutants are as efficient as the WT chaperone in preventing aggregation.

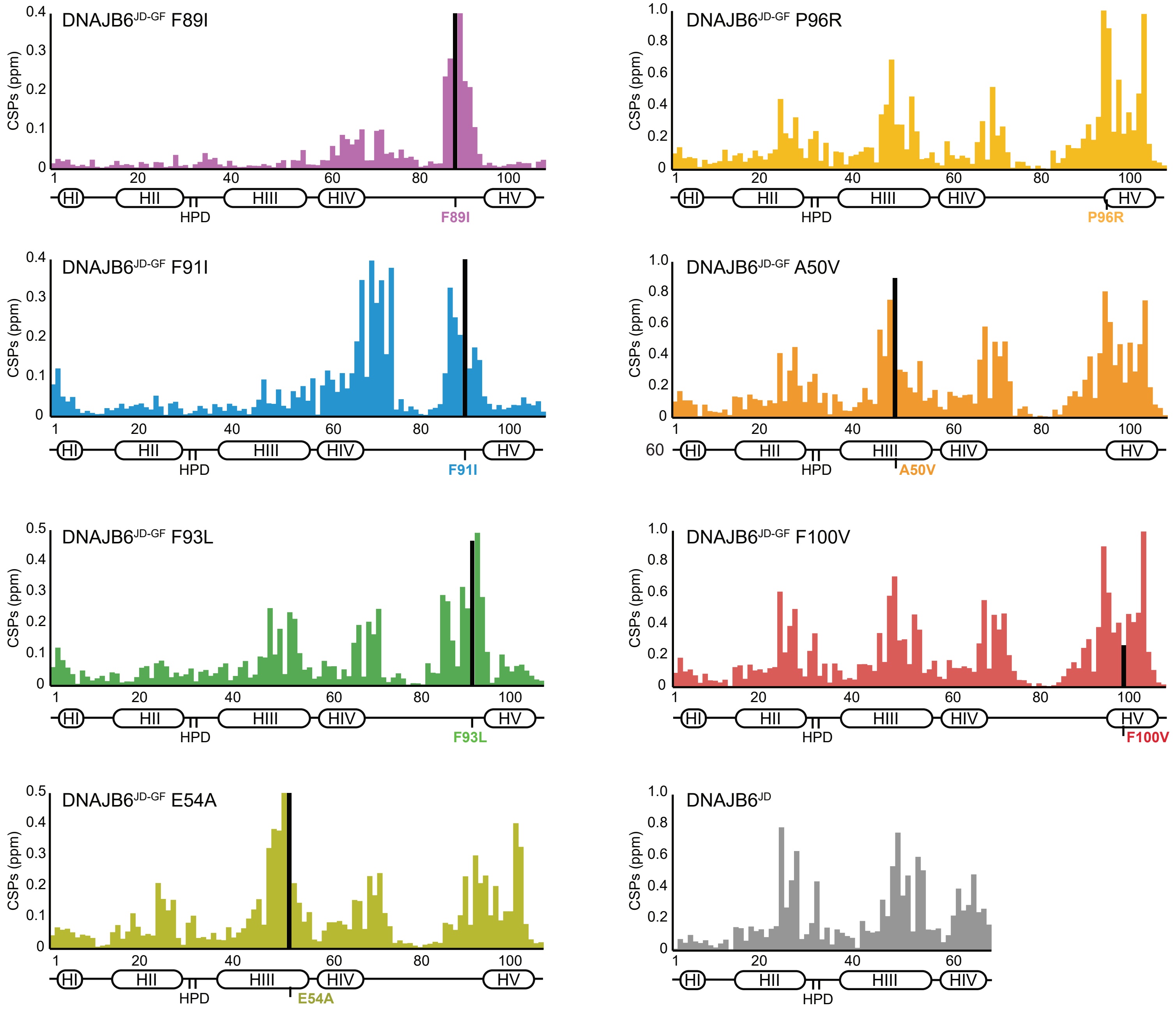

Supplementary Figure 2. LGMDD1 mutations disrupt the GF inhibition of the J-domain

Differences in chemical shift positions between DNAJB6$^{JD\text{-}GF}$ WT and LGMDD1 mutants. The differences in the chemical shift positions ($\Delta\delta$) are defined by the relation $\Delta\delta = \sqrt{(\Delta\delta_H)^2 + (\frac{\Delta\delta_N}{5})^2}$, where $\Delta\delta_H$ and $\Delta\delta_N$ are ^{1}H proton and ^{15}N nitrogen shift changes between the chemical shifts of WT and mutant DNAJB6 JD-GF residues (1-110). All mutants cause perturbations to helixes II and III, corresponding to the undocking of the GF helix V from the J-domain. The domain organization and the position of the LGMDD1 disease mutation is indicated at the top of each plot.

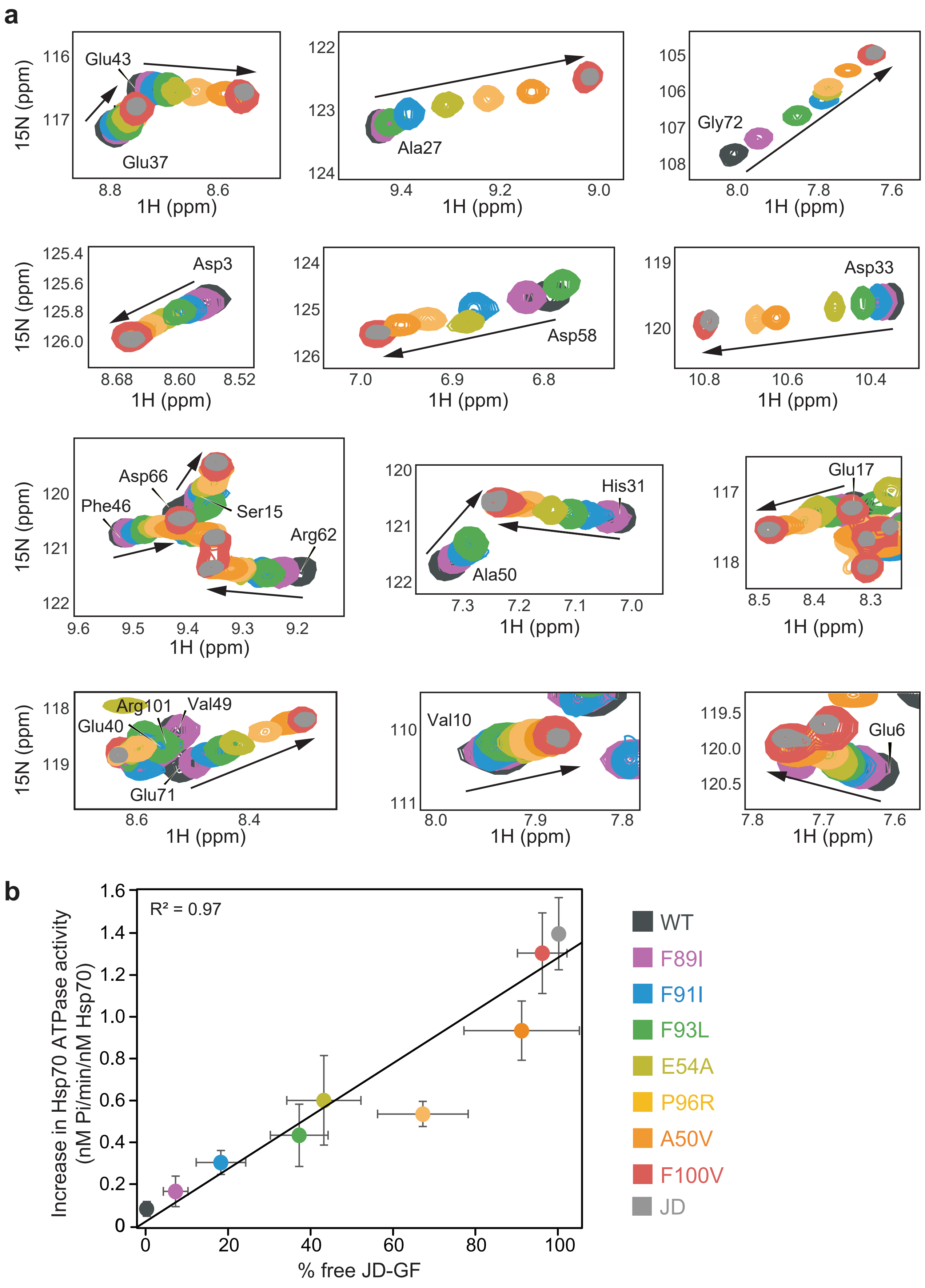

Supplementary Figure 3. LGMDD1 mutations disrupt the GF inhibition of the J-domain
(a) Superposition of selected ^{15}N–^{1}H HSQC spectral regions of WT (dark gray) and mutant DNAJB6$^{JD\text{-}GF}$ (colored as indicated) focusing on 21 residues displaying a progressive titration of cross-peak chemical shifts towards a conformation in which the GF is undocked from the J-domain. DNAJB6$^{1\text{-}96}$ variant lacking the inhibitory helix 5 is shown at lower contours to mark the position of the fully open DNAJB6$^{JD\text{-}GF}$ conformation (light grey). **(b)** Correlation between the DNAJB6-dependent increase in Hsp70 ATPase activity and the percentage of the free JD-GF population present in each DNAJB6$^{JD\text{-}GF}$ variant.

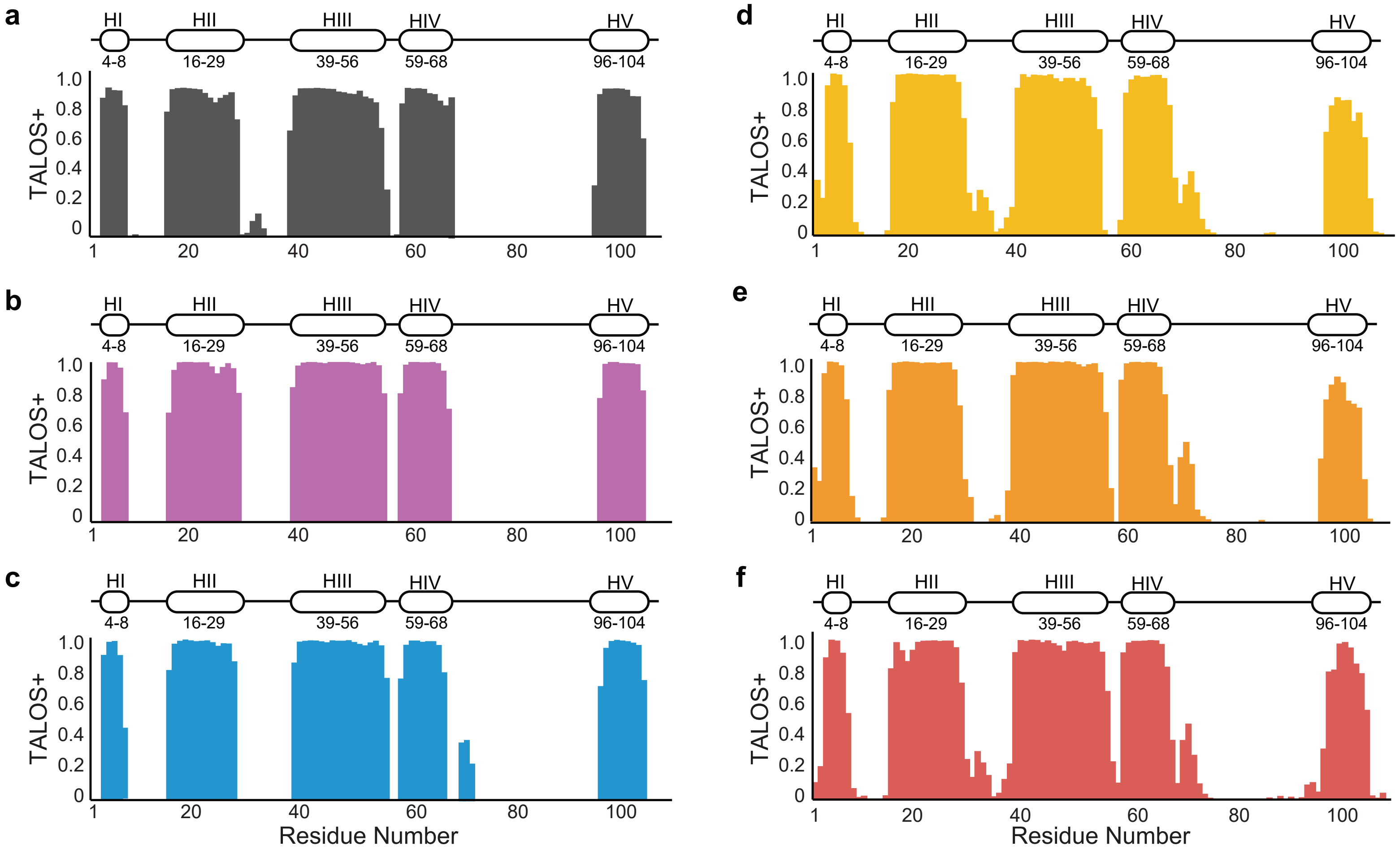

Supplementary Figure 4. GF helix V remains folded once undocked from the J-domain
(a-f) TALOS⁺ secondary structure probability derived from the backbone $^{13}C\alpha$, $^{13}C_{\beta}$, $^{13}C'$, ^{15}N, and $^{1}H_{N}$ chemical shifts for DNAJB6$^{JD\text{-}GF}$ WT (a; gray), F89I (b; purple), F91I (c; blue), P96R (d, yellow), A50V (e; orange) and F100V (f, red). Helix V remained folded in all tested DNAJB6$^{JD\text{-}GF}$ mutant constructs.

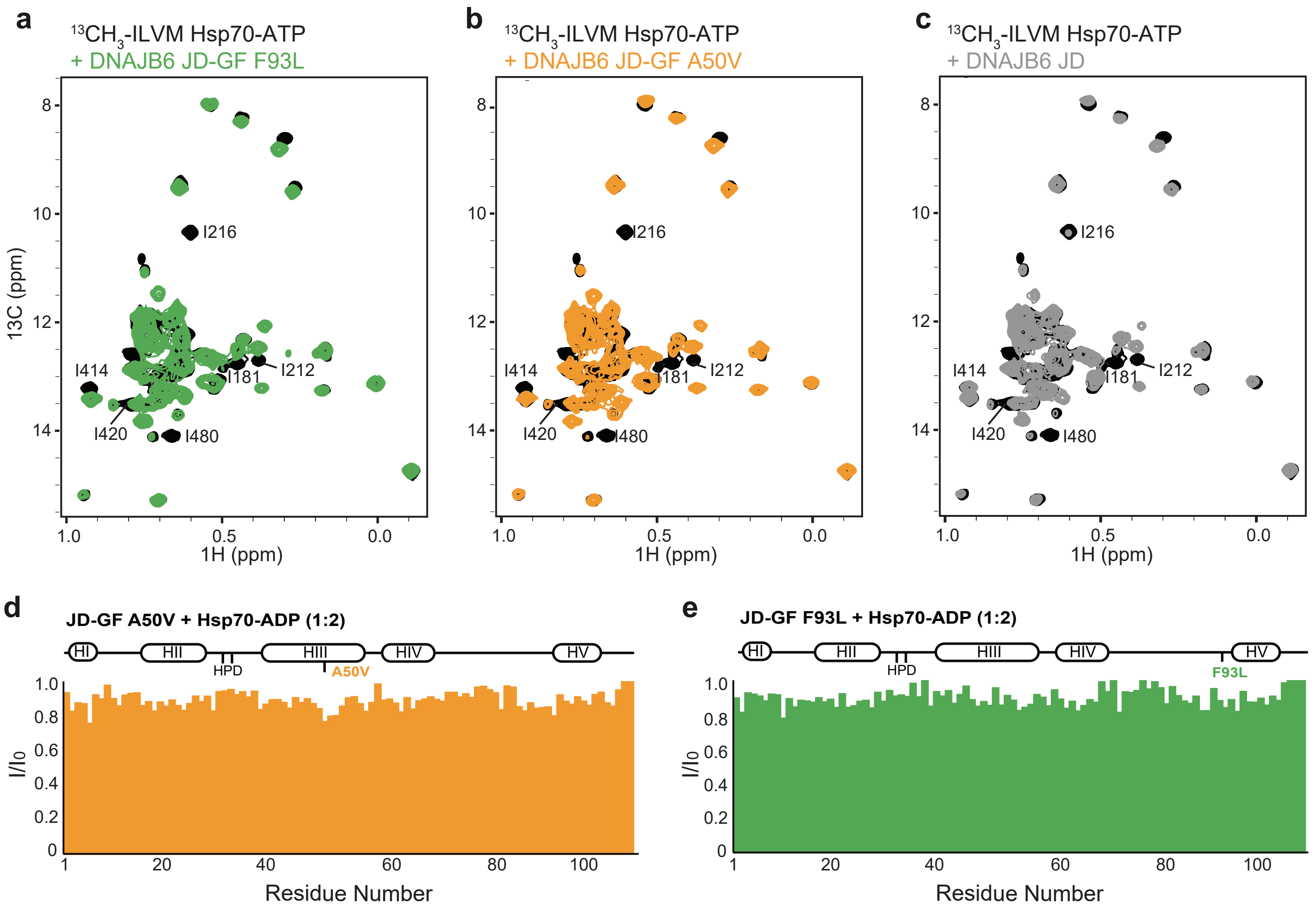

Supplementary Figure 5. The GF region of DNAJB6 does not interact directly with Hsp70
(a-c) Overlay of ^{1}H-^{13}C HMQC correlation maps of ILVM-labeled Hsp70 (black), and Hsp70 in complex with DNAJB6$^{JD\text{-}GF}$ F93L (a, green), A50V (b, orange), or DNAJB6^{JD} lacking the GF-region (c, light gray). Identical binding profiles are observed for both the mutants and the J-domain, confirming that the GF region does not form contacts with Hsp70. **(d-e)** Residue-resolved NMR signal intensity ratios I/I_0 for A50V (d) or F93L (e) DNAJB6$^{JD\text{-}GF}$ constructs upon addition of 2-fold molar excess of ADP-bound Hsp70. The positions of the four helices in each J-domain are indicated at the top of the plot. No changes in intensity are detected, indicating that Hsp70 does not interact with the GF region of DNAJB6 as a client protein.

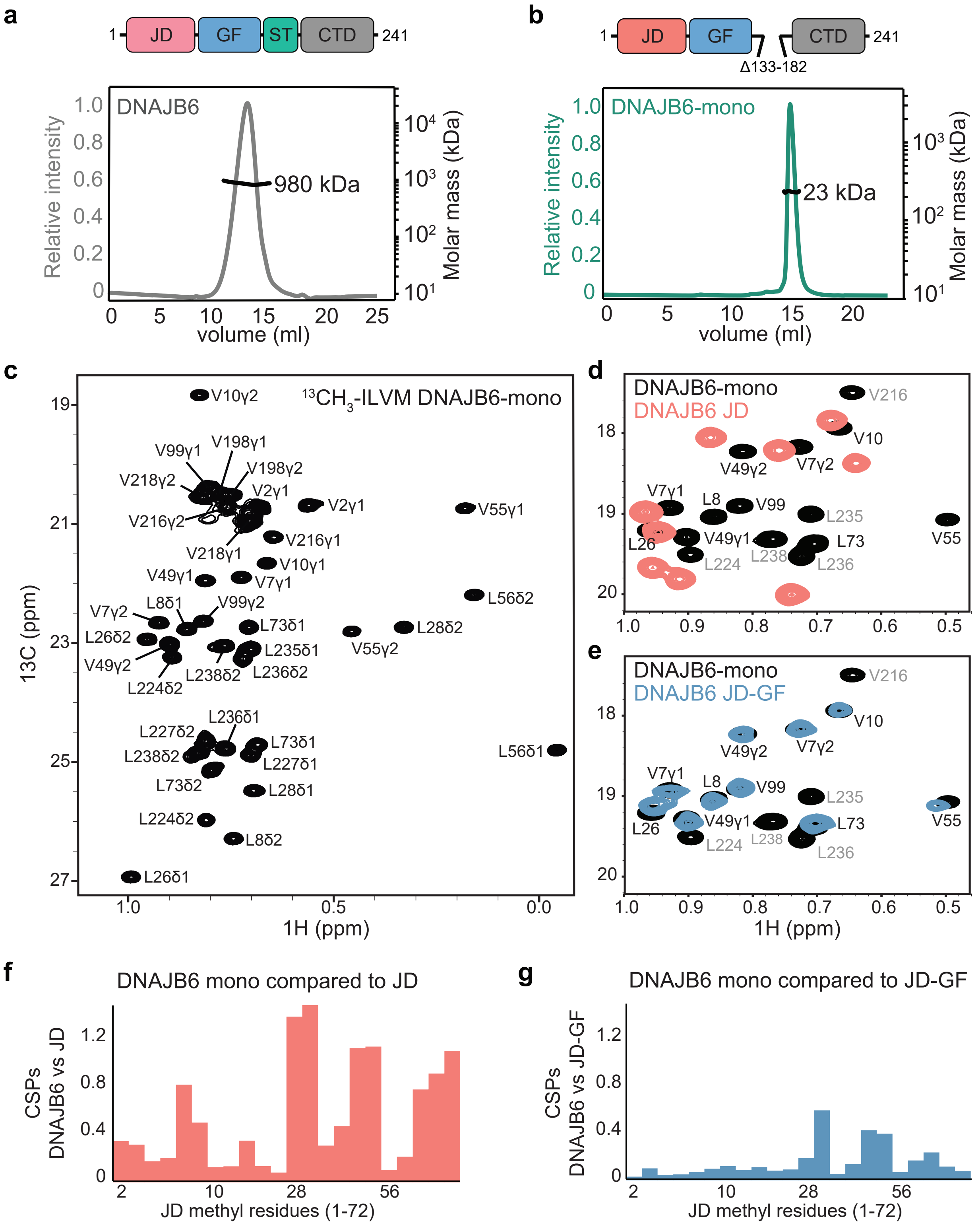

Supplementary Figure 6. Characterization of monomeric DNAJB6 (DNAJB6 ΔST)
(a) SEC-MALS analysis of full-length WT DNAJB6. The chaperone forms ~1MDa assemblies, corresponding to 36-mer homo-oligomers. Protein separation was performed on a Superose 6 column **(b)** SEC-MALS analysis of DNAJB6 lacking the ST region (DNAJB6 ΔST), separated on a Superdex 200 column. DNAJB6 ΔST assembles into stable monomeric complexes of 23 kDa. **(c)** ^{1}H-^{13}C HMQC spectrum of $^{13}CH_3$-ILVM labeled monomeric DNAJB6 ($DNAJB6^{mono}$). The assignments of the methyl residues [19] are indicated. **(d-e)** Overlay of ^{1}H-^{13}C HMQC spectra of $^{13}CH_3$-ILVM labeled $DNAJB6^{mono}$ (black) and $DNAJB6^{JD}$ (pink; d) or $DNAJB6^{JD\text{-}GF}$ (blue; e) constructs. The $DNAJB6^{JD\text{-}GF}$ peaks overlap mostly with those of full length DNAJB6, while those of free $DNAJB6^{JD}$ do not, indicating that in the full-length protein the J-domain is in the GF-inhibited conformation. **(f-g)** CSPs plot between $DNAJB6^{mono}$ and $DNAJB6^{JD}$ (pink; f) or $DNAJB6^{JD\text{-}GF}$ (blue; g) constructs. CSPs are defined by the relation $\Delta\delta = \sqrt{\left(\frac{\Delta\delta_H}{\alpha}\right)^2 + \left(\frac{\Delta\delta_C}{\beta}\right)^2}$, where $\Delta\delta_H$ and $\Delta\delta_C$ are methyl ^{1}H and ^{13}C chemical shift changes between full length and JD (f) or JD-GF (g) truncations of the protein, and α (β) is one standard deviation from the methyl ^{1}H (^{13}C) chemical shifts deposited in the Biological Magnetic Resonance Data Bank (α is 0.29 (I), 0.28 (L), 0.27 (V) and 0.41 (M), whereas β is 1.65 (I), 1.6 (L), 1.4 (V), and 1.54 (M)).

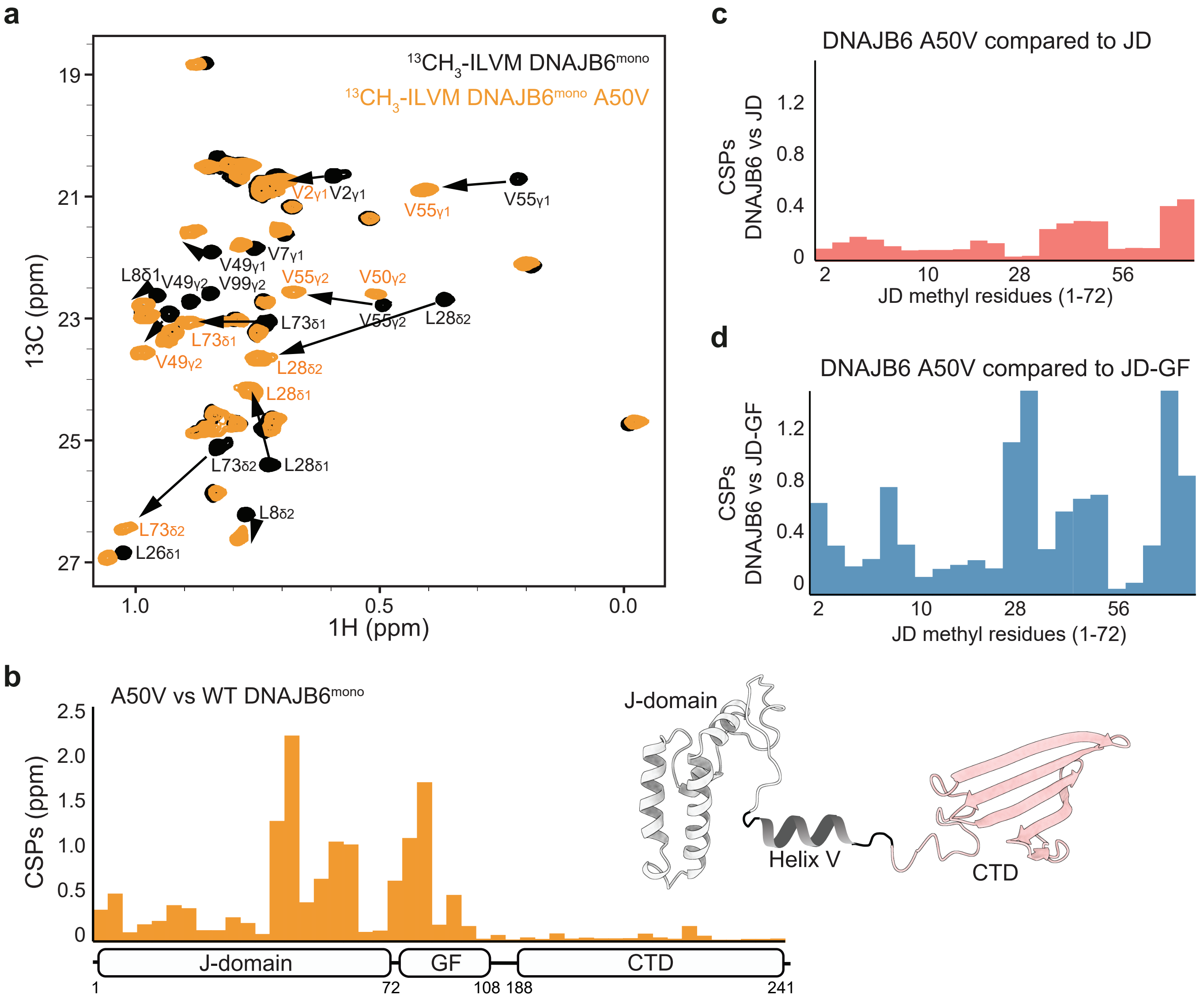

Supplementary Figure 7. DNAJB6 A50V LGMDD1 mutant

(a) Overlay of ^{1}H-^{13}C HMQC spectra comparing ^{13}CH$_3$-ILVM labeled WT DNAJB6^{mono} (black) and DNAJB6^{mono} A50V mutant (orange). **(b)** CSPs are primarily localized to the J-domain region, indicating a conformational change in the A50V mutant to undocked, free, J-domain. **(c-d)** CSPs plots comparing the DNAJB6^{mono} A50V mutant and DNAJB6^{JD} (c) or DNAJB6$^{JD\text{-}GF}$ (d) truncated constructs. The DNAJB6^{mono} A50V mutant peaks predominantly overlap with those of the free J-domain and not with the inhibited JD-GF. CSPs are defined by the relation -

$\Delta\delta = \sqrt{\left(\frac{\Delta\delta_H}{\alpha}\right)^2 + \left(\frac{\Delta\delta_C}{\beta}\right)^2}$, where $\Delta\delta_H$ and $\Delta\delta_C$ are methyl ^{1}H and ^{13}C chemical shift changes between full length and JD (f) or JD-GF (g) truncations of the protein, and α (β) is one standard deviation from the methyl ^{1}H (^{13}C) chemical shifts deposited in the Biological Magnetic Resonance Data Bank (α is 0.29 (I), 0.28 (L), 0.27 (V) and 0.41 (M), whereas β is 1.65 (I), 1.6 (L), 1.4 (V), and 1.54 (M)).

a

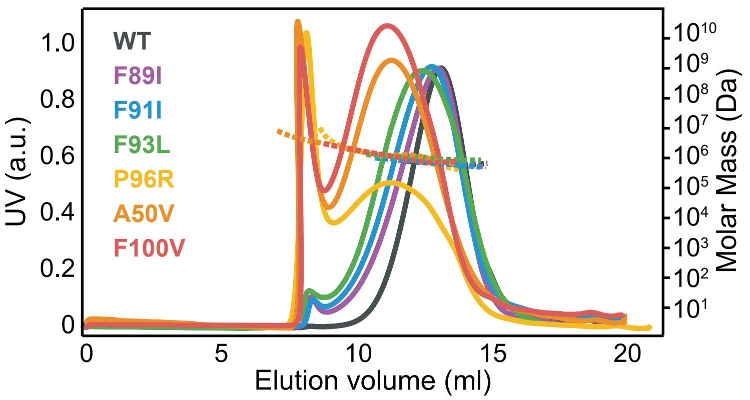

b

DNAJB6 variant	MW (KDa)	Tm (°C)
WT	920 ± 70	68.3 ± 0.4
F89I	950 ± 140	70.4 ± 0.6
F91I	1020 ± 200	70.0 ± 0.4
F93L	1430 ± 180	68.4 ± 0.6
P96R	1220 ± 230	67.1 ± 0.6
A50V	1460 ± 260	68.2 ± 0.4
F100V	1240 ± 340	67.1 ± 0.6

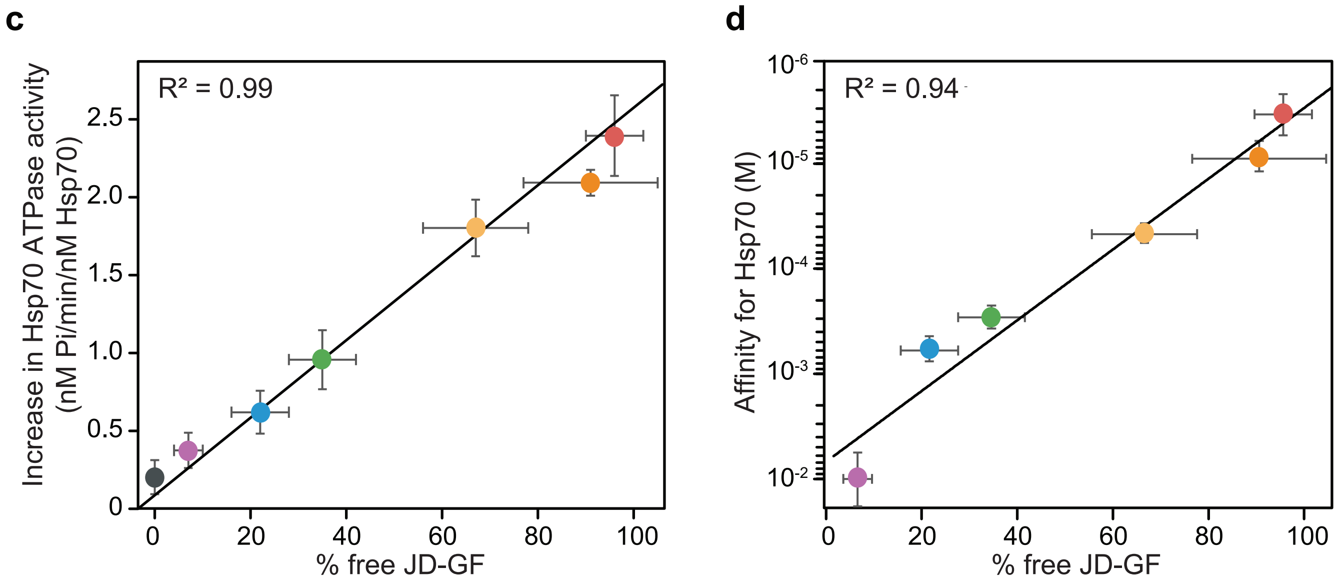

Supplementary Figure 8. DNAJB6 mutants display unregulated interaction with Hsp70 (a) SEC-MALS analysis of full-length oligomeric WT DNAJB6 and LGMDD1 associated mutants separated on a Superose 6 column. Despite exhibiting different elution profiles, all pathogenic mutants assemble into high-order oligomers similar in size to the WT protein. LGMDD1 mutants with a high population of free J-domain elute at earlier fractions, indicating a higher radius of gyration, likely due to J-domain detachment. **(b)** Table summarizing the calculated molecular weights and melting temperatures of WT DNAJB6 and LGMDD1-associated mutants. All variants have similar T_ms and assemble into ~1 MDa assemblies corresponding to ~36 monomeric DNAJB6 units. **(c)** Correlation between the DNAJB6-dependent increase in Hsp70 ATPase activity and the percentage of the free JD-GF population present in each DNAJB6 LGMDD1 disease mutant. Data are presented as mean values +/- SEM. **(d)** Correlation between the affinity of DNAJB6 to Hsp70 (in M) and the percentage of the free JD-GF population present in each DNAJB6 variant. Data are presented as mean values +/- SEM.

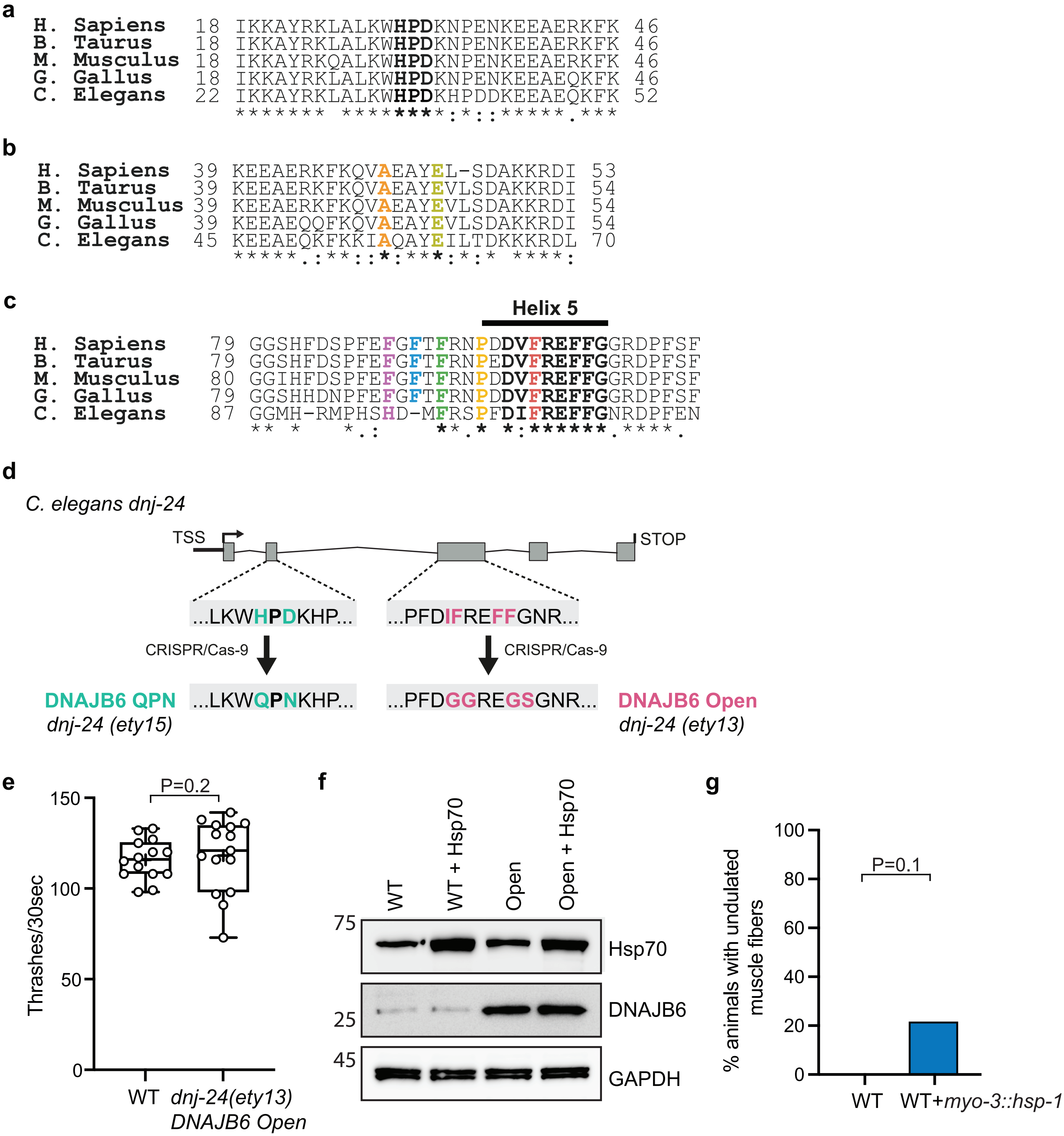

Supplementary Figure 9. DNAJB6 mutants display toxic gain of function through unregulated interaction with Hsp70 chaperones

(a-c) Multiple species sequence alignment highlighting the conservation of the J-domain and the inhibitory GF helix V across species. The HPD motif and the inhibitory helix V are indicated in bold. Residues frequently mutated in LGMDD1 patients are color-coded. Asterisks indicate conserved amino acids at each position. **(d)** CRISPR-Cas9-mediated mutagenesis of the *dnj-24* HPD motif and the inhibitory helix V. Genomic nucleotide changes are indicated in magenta (mutations to the inhibitory helix V, "Open"), and cyan (mutations to HPD motif- QPN). **(e)** Quantification of a thrashing assay carried out on wild-type and DNAJB6^{Open} mutant animals. Statistics were computed using Mann-Whitney test, two-tailed. Number of animals- Wild-type, n=14; DNAJB6^{Open}, n=15. **(f)** Western blots showing the protein levels of HSP-1/Hsp70 and DNJ-24/DNAJB6 in *C. elegans* strains expressing WT DNAJB6 (DNJ-24) or DNAJB6^{Open} mutant, with or without muscle-specific overexpression of Hsp70. **(g)** Quantification of animals displaying the undulated muscle phenotype in strains expressing WT DNAJB6 (DNJ-24) and in the absence or presence of muscle specific overexpression of Hsp70 (*myo-3::hsp-1*). Statistics is Fisher's exact test, two tailed. n=20 for each group.

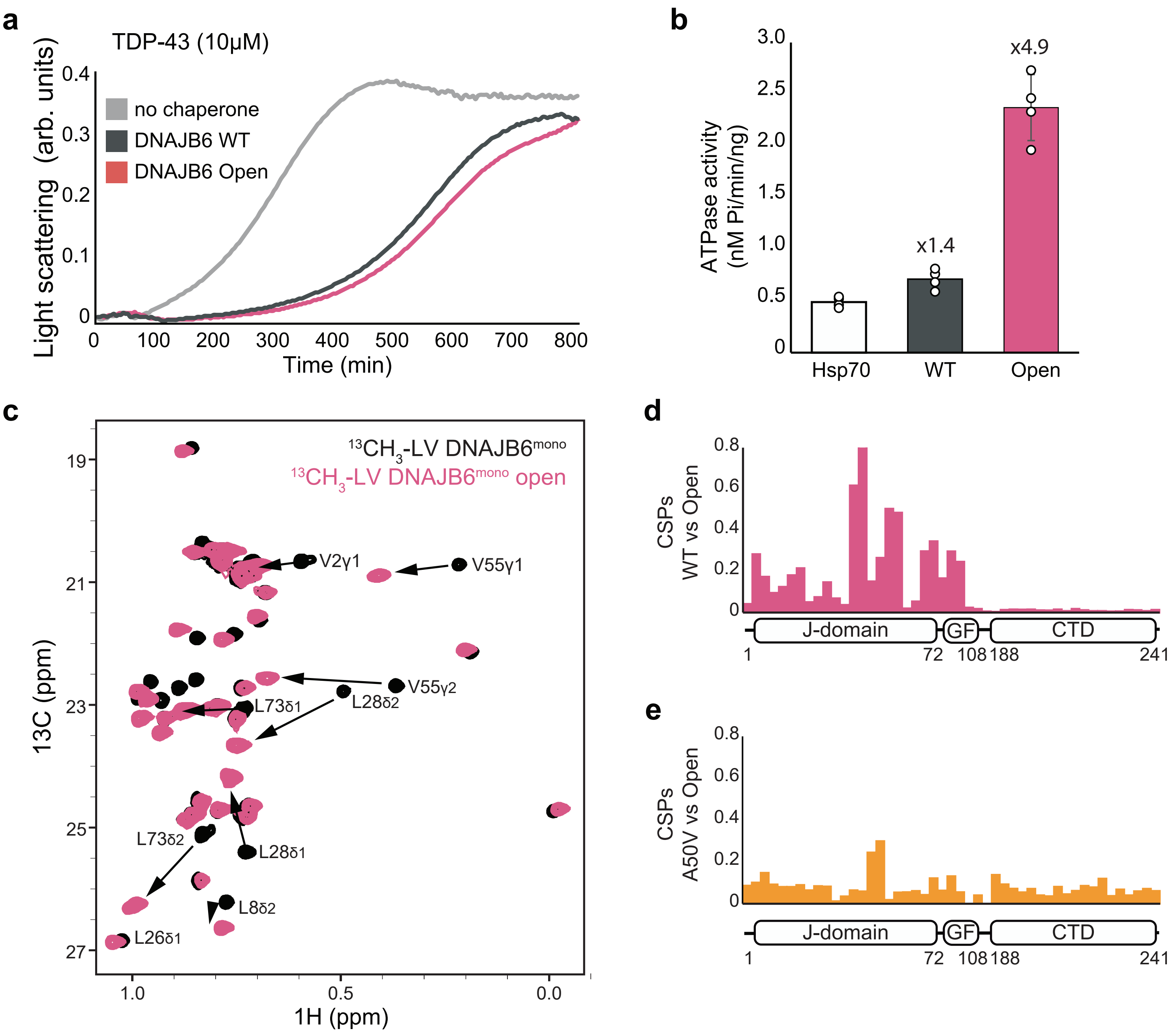

Supplementary Figure 10. DNAJB6 mutant with artificially released JD-GF inhibition

(a) Aggregation of 10 µM TDP-43 alone (light gray) and in the presence of 5 µM of WT DNAJB6 (dark gray) or DNAJB6^{Open} mutant (magenta). The mutant is as efficient as the WT chaperone in preventing aggregation. **(b)** Steady state ATPase activity of Hsp70 alone (white), and Hsp70 incubated with WT DNAJB6 (gray) or DNAJB6^{Open} mutant (magenta). The DNAJB6^{Open} mutant, which lacks the JD-GF inhibition, enhances Hsp70 ATP hydrolysis rates 5-fold, while only a minor, 1.4-fold activation was detected upon addition of WT DNAJB6 to Hsp70. Data are means ± SEM ($n = 4$ independent experiments). **(c)** Overlay of ^{1}H-^{13}C HMQC spectra comparing $^{13}CH_3$-ILVM labeled WT DNAJB6 (black) and DNAJB6^{Open} mutant (magenta) which artificially releases the JD-GF inhibition. **(d)** CSPs plots comparing DNAJB6^{Open} mutant and DNAJB6 WT, showing large changes in the J-domain region **(e)** CSPs plots comparing DNAJB6^{Open} and DNAJB6^{mono} A50V mutant. Only small CSPs are detected, mainly near the site of the mutation, indicating that DNAJB6^{Open} adopts a similar uninhibited conformation as the LGMDD1 mutants.
