# Supplementary material for: DNAJB6 mutants display toxic gain of function through unregulated interaction with Hsp70 chaperones
Source: Nat Commun. 2023 Nov 3;14:7066. doi: 10.1038/s41467-023-42735-z (PMC10624832; doi:10.1038/s41467-023-42735-z)
Supplement: Supplementary file 3 — Description of Additional Supplementary Files [file 41467_2023_42735_MOESM3_ESM.docx]

**File Name:** Supplementary Data 1

**Description:** The top 10 models for DNAJB6^JD^ (residues 1-72) modelled using CS-Rosetta.
