# Supplementary material for: DNAJB6 mutants display toxic gain of function through unregulated interaction with Hsp70 chaperones
Source: Nat Commun. 2023 Nov 3;14:7066. doi: 10.1038/s41467-023-42735-z (PMC10624832; doi:10.1038/s41467-023-42735-z)
Supplement: Supplementary file 5 — Reporting Summary [file 41467_2023_42735_MOESM5_ESM.pdf]

nature portfolio

Corresponding author(s): Rina Rosenzweig

Last updated by author(s): Oct 9, 2023

Reporting Summary

Nature Portfolio wishes to improve the reproducibility of the work that we publish. This form provides structure for consistency and transparency in reporting. For further information on Nature Portfolio policies, see our Editorial Policies and the Editorial Policy Checklist.

Statistics

For all statistical analyses, confirm that the following items are present in the figure legend, table legend, main text, or Methods section.

n/a	Confirmed	
☐	☒	The exact sample size (*n*) for each experimental group/condition, given as a discrete number and unit of measurement
☐	☒	A statement on whether measurements were taken from distinct samples or whether the same sample was measured repeatedly
☐	☒	The statistical test(s) used AND whether they are one- or two-sided<br>*Only common tests should be described solely by name; describe more complex techniques in the Methods section.*
☒	☐	A description of all covariates tested
☒	☐	A description of any assumptions or corrections, such as tests of normality and adjustment for multiple comparisons
☐	☒	A full description of the statistical parameters including central tendency (e.g. means) or other basic estimates (e.g. regression coefficient) AND variation (e.g. standard deviation) or associated estimates of uncertainty (e.g. confidence intervals)
☐	☒	For null hypothesis testing, the test statistic (e.g. *F*, *t*, *r*) with confidence intervals, effect sizes, degrees of freedom and *P* value noted<br>*Give P values as exact values whenever suitable.*
☒	☐	For Bayesian analysis, information on the choice of priors and Markov chain Monte Carlo settings
☒	☐	For hierarchical and complex designs, identification of the appropriate level for tests and full reporting of outcomes
☒	☐	Estimates of effect sizes (e.g. Cohen's *d*, Pearson's *r*), indicating how they were calculated

Our web collection on statistics for biologists contains articles on many of the points above.

Software and code

Policy information about availability of computer code

Data collection	NMR data were collected on Bruker spectrometers operated with TOPSPIN 3.7-4.2
Data analysis	NMR data were processed with NMRPipe and analyzed with CcpNmr Analysis version 3 and Sparky.

For manuscripts utilizing custom algorithms or software that are central to the research but not yet described in published literature, software must be made available to editors and reviewers. We strongly encourage code deposition in a community repository (e.g. GitHub). See the Nature Portfolio guidelines for submitting code & software for further information.

Data

Policy information about availability of data

All manuscripts must include a data availability statement. This statement should provide the following information, where applicable:

- Accession codes, unique identifiers, or web links for publicly available datasets
- A description of any restrictions on data availability
- For clinical datasets or third party data, please ensure that the statement adheres to our policy

NMR chemical shifts have been deposited in the Biological Magnetic Resonance Data Bank (BMRB) under the following accession codes: 51996 for DnaJB6JD , 52098 for DNAJB6JD-GF F100V, 51997 for DNAJB6JD-GF A50V , 51998 for DNAJB6JD-GF P96R , 52091 for DNAJB6JD-GF F91I , and 52065 for DNAJB6JD-GF F89I. The protein structure of DNAJB6 used for analysis in this study is available in the Protein Data Bank under accession code 6U3R. Data supporting the findings of this study are available in the Supplementary Information file. Source data are provided with this paper.

Research involving human participants, their data, or biological material

Policy information about studies with human participants or human data. See also policy information about sex, gender (identity/presentation), and sexual orientation and race, ethnicity and racism.

Reporting on sex and gender	n/a
Reporting on race, ethnicity, or other socially relevant groupings	n/a
Population characteristics	n/a
Recruitment	n/a
Ethics oversight	n/a

Note that full information on the approval of the study protocol must also be provided in the manuscript.

Field-specific reporting

Please select the one below that is the best fit for your research. If you are not sure, read the appropriate sections before making your selection.

☒ Life sciences ☐ Behavioural & social sciences ☐ Ecological, evolutionary & environmental sciences

For a reference copy of the document with all sections, see nature.com/documents/nr-reporting-summary-flat.pdf

Life sciences study design

All studies must disclose on these points even when the disclosure is negative.

Sample size	Sample sizes were selected according to literature showing similar methods of analysis: doi.org/10.1007/s12192-013-0448-5, doi.org/10.7554/eLife.69601, and doi.org/10.1073/pnas.95.26.15253.
Data exclusions	No data was excluded from the analyses. Sample sizes were selected according to literature showing similar methods of analysis:
Replication	Experiments were replicated to ensure reproducibility of the findings. The number of independent replicates for each experiment are specified in the respective Figure captions. All attempts at replication were successful.
Randomization	No grouped samples
Blinding	No grouped samples

Reporting for specific materials, systems and methods

We require information from authors about some types of materials, experimental systems and methods used in many studies. Here, indicate whether each material, system or method listed is relevant to your study. If you are not sure if a list item applies to your research, read the appropriate section before selecting a response.

Materials & experimental systems

n/a	Involved in the study	
☐	☒	Antibodies
☒	☐	Eukaryotic cell lines
☒	☐	Palaeontology and archaeology
☐	☒	Animals and other organisms
☒	☐	Clinical data
☒	☐	Dual use research of concern
☒	☐	Plants

Methods

n/a	Involved in the study	
☒	☐	ChIP-seq
☒	☐	Flow cytometry
☒	☐	MRI-based neuroimaging

Antibodies

Antibodies used	The following antibodies were used: Hsp70 (3A3, ab5439, Abcam, 1:5000), GAPDH (G8795, Sigma-Aldrich, 1:1000), goat anti mouse

Antibodies used	IgGHRP (JIR 115-035-003), and goat anti rabbit IgG-HRP (JIR 111-035-003).
Validation	All antibodies were validated for the species and immunoblotting by the manufacturer, as described on the manufacturer's website.

Animals and other research organisms

Policy information about studies involving animals; ARRIVE guidelines recommended for reporting animal research, and Sex and Gender in Research

Laboratory animals	C. elegans, N2 Bristol strain. All the strains used in this study are listed in the methods section. Experiments were performed at 1 day adult stage.
Wild animals	none
Reporting on sex	Data was taken from hermaphrodites, males were analyzed and exhibited similar phenotypes
Field-collected samples	No field collected studies were used in this study
Ethics oversight	Ethical approval is not required for work with soil nematodes

Note that full information on the approval of the study protocol must also be provided in the manuscript.
